# Cellular and Molecular Mechanisms in Neurodevelopmental Disorders and Brain Tumors

**DOI:** 10.3390/ijms24119469

**Published:** 2023-05-30

**Authors:** Mario Costa, Eleonora Vannini

**Affiliations:** 1 Neuroscience Institute, National Research Council (CNR), Via G. Moruzzi 1, 56124 Pisa, Italy; 2 Centro Pisano Ricerca e Implementazione Clinica Flash Radiotherapy “CPFR@CISUP”, “S. Chiara” Hospital, 56124 Pisa, Italy; 3 Laboratory of Biology BIO@SNS, Scuola Normale Superiore, Piazza di Cavalieri 7, 56124 Pisa, Italy

The normal growth and operation of the central nervous system (CNS) at all stages of development, including adulthood, depend on the interaction between intrinsic and extrinsic factors. Indeed, any deviation from normal CNS function due to various extrinsic factors, such as social deprivation, environmental influences, infectious diseases, or nutritional factors, may result in abnormal neuronal architecture or connectivity and eventually neurodevelopmental disorders. In this extremely dynamic scenario, it has also been shown that intrinsic genetic deregulation of certain protein dosages or uncontrolled timing of gene expression can lead to cancer and/or neurodevelopmental disorders. On the other hand, individuals with certain neurodevelopmental disorders, such as autism, may have an increased risk of developing cancer [1]. Transcription factors are known to play a crucial role in regulating gene expression, and their deregulation, including that triggered by viral infections, has been associated with tumors and neurodevelopmental disorders [2]. For instance, mutations in MeCP2 or FOXG1, two transcription factors that are able to impact Wnt/β-catenin signaling, have been implicated in both conditions, i.e., in neurodevelopmental disorders and cancer [3,4,5]. Overall, these puzzling observations highlight the complex relationship between cancer and neurodevelopmental disorders. 

Gliomas are aggressive tumors of the central nervous system that are characterized by genetic alterations and deregulated signaling pathways. Christina Piperi’s group describes a selection of oncogenic (GLI-1/2/3, E2F1–8, STAT3, and HIF-1/2) and tumor suppressor (NFI-A/B, TBXT, MYT1, and MYT1L) transcription factors that are deregulated in gliomas and associated with tumor development [6]. Among the oncogenic transcription factors described, it is worth highlighting that GLI proteins, involved in glioma growth, are downstream effectors of the Sonic Hedgehog pathway. Nevertheless, mutations in the SHH pathway are associated with various neurodevelopmental disorders, including Rett and FOXG1 syndromes in humans. Similarly, tumor suppressor transcription factors such as NFI-A/B, TBXT, MYT1, and MYT1L, which are frequently downregulated in gliomas, are also key players in the physiopathology of neurodevelopment. For example, TBXT is involved in embryonic development and has been shown to inhibit glioma growth, while MYT1 and MYT1L transcription factors regulate the differentiation of neural progenitor cells and are frequently lost in gliomas. In this review, Giannopoulou et al. [6] discuss the current options to target these transcription factors with bortezomib, a drug able to induce multiple myeloma osteoblast differentiation via Wnt-independent activation of β-catenin signaling, and natural plumbagin, which is able to affect the Wnt/β-catenin pathway and inhibit cancer stem-like cells by suppressing cell proliferation and invasion. So and colleagues, in their research [7], proved that knockout animal models for the microglial fatty-acid binding protein FABP4 display changes in the hippocampal transcriptome, suggesting that FABP4 is a potential target in alleviating high-fat diet-induced neuroinflammation and cognitive decline, highlighting the role of WNT/β-catenin in this protection. However, potential therapeutic targeting of FABP4 or the WNT/β-catenin pathway would need to consider potential risks and benefits in the context of cancer progression and brain development. 

The role of the human microbiome in both cancer development and autism spectrum disorders (ASD) is now clear. However, although several aspects linking the brain-gut axis with these two disorders have been clarified, i.e., the relations with the immune system or endocrine mediators, further research is necessary to determine a specific shared signature for both pathologies. Kraneveld’s group [8] investigates the role of p-cresyl sulfate (pCS), a bacterial metabolite found in elevated concentrations in the urine and feces of children with autism spectrum disorders. Their work shows that ADAM10 and ADAM17 play partial but distinct roles in disrupting the innate immune response of microglial cells associated with pCS-induced ASD pathogenesis in the LPS neuroinflammation model [8]. Mansouri and colleagues, on the other hand, [9] examined the autism rat model and found that while environmental enrichment significantly ameliorated repetitive behavior triggered by maternal separation, it also negatively affected anxiety and social behavior, which may be modulated, as suggested in the paper, by plasma BDNF levels.

Takata and colleagues [10] performed DNA microarray analysis and quantitative RT-PCR on the fetal cerebrum after maternal intraperitoneal or fetal intracerebral ventricular injection of leukemia inhibitory factor (LIF). Indeed, LIF is involved in cerebral development via the placenta in rat models, and maternal immune activity is connected to psychiatric problems in children. In this report, the authors demonstrate increased levels of IGF-1 and IGF-2 in fetal cerebrospinal fluid (CSF). Importantly, the authors claimed that LIF treatment via IGF-1 and IGF-2 promotes proliferation of neuronal progenitor cells, suggesting the involvement of the LIF–IGF axis in developing the cerebral cortex. In light of this, the findings obtained in the rodent animal model on the LIF-IGF axis represent significant breakthroughs in understanding the developmental origins of health and disease associated with psychiatric disorders in children.

NOTCH3 signaling is implicated together with other genes in vasculogenesis, Cerebral Autosomal Dominant Arteriopathy with Subcortical Infarcts, and Leukoencephalopathy (CADASIL), a hereditary disease associated with migraines, psychiatric disorders, recurrent stroke, and dementia. This review showed a clear panorama of potential key pathways related to CASIDIL progression and could be useful for researchers to understand the biological mechanisms and find potential drug targets for CADASIL [11].

In conclusion, this selection of papers will hopefully encourage further studies to elucidate some of the underlying common mechanisms in cancer and neurodevelopmental disorders.
